# For the Complexion

**Published:** 1893-06

**Authors:** 


### FOR THE COMPLEXION.

Wash your own dishes, polish your own brass and silver, sweep and dust, and make up your own bed, water and tend your own flowers; in fact, keep yourself busy and in good spirits, or take a brisk walk or ride in the afternoon of each day in pretty weather. Eat eggs, milk, rare steaks, wild meats, and other digestible food, leaving off everything fried, rich in condiments and fats. Sleep seven or eight hours in the twenty-four, in a well ventilated room, in which the sun has been permitted to shine two hours each day. Let the light fall on you; you are like a plant, you need it. And in less than a year your complexion will be better than any lotion or pomade could make it.—*Ex.*

